# Different Types of Visual Perturbation Induced Different Demands and Patterns in Active Control: Implication for Future Sensorimotor Training

**DOI:** 10.3389/fphys.2022.919816

**Published:** 2022-07-13

**Authors:** Muchen Ren, Tangdi Lin, Jung Hung Chien

**Affiliations:** ^1^ Department of Health and Rehabilitation Science, College of Allied Health Professions, University of Nebraska Medical Center, Omaha, NE, United States; ^2^ Independent Researcher, Omaha, NE, United States

**Keywords:** optic flow, virtual reality, visual perturbation, active control, treadmill walking

## Abstract

**Background:** Sensorimotor training using visual perturbations has been widely applied to astronauts for rapidly handling and adapting to unpredictable environments. However, these visual perturbations might not be strong enough to trigger long-term effects. Therefore, this study aimed to develop a novel sensorimotor training paradigm using pseudo-random visual perturbations and to determine the demands and patterns of active control under different types of visual perturbations.

**Method:** Thirty healthy young adults participated in this study. Four walking conditions were randomly assigned to these participants: 1) walking without optic flow (NoOptic), 2) walking with the optic flow (Optic), 3) walking under reduced visual capability (Vre), and 4) walking under perturbed optic flow (Vpe). The dependent variables were the step length variability, the step width variability, the 95% confidence interval ellipse area, the long axis of the ellipse, and the short axis of the ellipse.

**Results:** The results indicated that 1) the step length variability and the ellipse area were greater in Vre compared to Optic (*p* < 0.001, *p* < 0.001). Moreover, the step width variability and ellipse area were greater in Vpe than Optic (*p* < 0.001, *p* = 0.002).

**Conclusion:** The abovementioned results demonstrated that 1) walking in both Vre and Vpe conditions required greater demands and different patterns in active controls compared to the Optic condition, suggesting both Vre and Vpe conditions could be applied for sensorimotor training; 2) the Vre condition would be the first choice if there were no concerns in potential trips on the treadmill.

## Introduction

In 1994, the National Aeronautics and Space Administration announced the start of the Mars Exploration Program. Since then, preparing for human exploration has never stopped, and allowing a human to walk on the surface of Mars has been a developing dream. However, rapidly adapting from one level of gravity to another level of gravity becomes a critical issue. Studies have observed the deterioration of motor control in astronauts after they return to the Earth, including the disruption in spatial orientation during walking (Glasauer et al., 1995), pattern changes in lower extremity kinematics (Courtine et al., 2002; Bloomberg and Mulavara, 2003; Miller et al., 2010), and reduction in visual acuity during walking (Miller et al., 2010). These deteriorations of motor control are highly related to the alternations in the vestibular system (Miller et al., 2010). Staying in a zero- or microgravity environment for a period of time leads to the adaptation of the vestibular system. Thus, when astronauts come back to the Earth, the vestibular system needs to be re-adapted to accommodate with Earth’s gravity (Carriot et al., 2021). At this moment, due to the adaptation of the vestibular system, astronauts would experience some locomotor instability and need other sensory systems, such as visual and somatosensory systems, to counter this instability. The average time of adaptation of gait from one level of gravity to another level of gravity takes approximately 15 days or above (Wood et al., 2011). These postflight compromised motor controls are expected to be observed when landing on Mars after a long space trip in the future. These results may hinder the crew members from executing the missions immediately after landing on Mars. Thus, in the past decades, sensorimotor training, which teaches humans to solve sensory-conflicted situations, has been developed. The core concept of this sensorimotor training is to challenge these astronauts to select appropriate locomotor strategies rapidly in unrehearsed and untrained environments.

The visual system is the dominant sensory system during walking, and high visual dependence for movement has been documented in astronauts (Young and Shelhamer, 1990). Therefore, walking under visual manipulations is one of the common sensorimotor trainings, including using visual-distorted lenses (Roller et al., 2001), artificial optic flow perturbations (Prokop et al., 1997; O’Connor and Kuo, 2009; McAndrew Young et al., 2012; Eikema et al., 2016), rotational optic flow (Berard et al., 2011), and visually polarized perturbations (Davids et al., 2003; Caballero et al., 2019). Among these studies, artificial optic flow has been widely applied in movement analysis laboratories. For instance, increasing the speed of optic flow decreases the walking speed on the treadmill (Prokop et al., 1997). Adding optic flow to split-belt treadmill walking enhances the effect of adaptation (Eikema et al., 2016). Manipulating the optic flow speed increases the level of active control and the margin of stability in the mediolateral-lateral direction (O'Connor and Kuo, 2009; McAndrew Young et al., 2012). In short, the purpose of these different types of visual perturbations is to enhance the capability of integrating reliable sensory systems to make an appropriate movement. However, the limitation of these studies is that these patterns of optic flow may not be “unexpected” enough for long-term training. The patterns of optic flow from the above studies are composed of different equations of the sinusoidal wave; therefore, the pattern of optic flow may be easily identified by human perception, although these studies indeed observe some significant changes in locomotor control, such as spatial-temporal gait parameters and respective variability. However, some subtle alternations of locomotor control due to the rhythmic visual perturbations may be adapted after a couple of steps on the treadmill. Thus, one of the goals of this study was to develop an “unexpected-enough” pattern of optic flow to challenge locomotor control.

The movement variability (standard deviation/coefficient of variation) of dependent variables has widely been used in the abovementioned studies to determine motor control under different sensory-conflicted conditions. Higher movement variability does not necessarily mean bad motor behavior; instead, this increase in movement variability plays a critical functional role in driving motor behaviors, allowing the central nervous system to make full use of the available sensory systems to explore and adapt to unfamiliar environments (Davids et al., 2003). For instance, Caballero et al.’s (Caballero et al., 2019) study, which asks young participants to walk a track with three different task constraints (track width, surface stiffness, and walking direction), observes the significant increase in movement variability when walking on the foam surface. The authors suggest that this increment of movement variability is attributed to a demand for perceiving enough environmental information for the central nervous system to appropriately regulate its movements (Caballero et al., 2019). Moreover, this abovementioned demand can be directional-dependent (O'Connor and Kuo, 2009), indicating that 1) different types of motor behaviors (e.g., standing and walking) require different demands in motor control, and 2) based on the natural human anatomical structure, the demands in motor control are less in the anterior-posterior (AP) than in the medial-lateral directions (ML), which terms the hypothesis of active lateral control (Bauby and Kuo, 2000). This active control further determines that when walking on the track/treadmill, the demands of motor control in the medial-lateral direction requires more than in the anterior-posterior direction when encountering the perturbations (O'Connor and Kuo, 2009). This concept of active control is further extended by a study, indicating that not only the demands in the active control but also the patterns in the active control need to be considered (Hu and Chien, 2021). In this previous study, ageing increases the demands of active control but selects the conservative pattern of active control during treadmill walking when both visual and somatosensory systems are perturbed. Differentiating the patterns of active control is crucial for developing further sensorimotor adaptability training (Hu and Chien, 2021).

This study attempted to extend the knowledge from the abovementioned studies and had one major aim: to develop a novel sensorimotor training paradigm for determining the demands and patterns of active control under different types of visual perturbations. This study hypothesized that 1) either perturbing or reducing optic flow would increase the demands of active control, and 2) different types of visual perturbations would change the patterns of active control differently.

## Materials and Methods

### Participants

A total of thirty young adults participated in this study (sixteen males and fourteen females; age 25.01 ± 5.21 years old, height 1.70 ± 0.43 m, and mass 68.03 ± 13.5 kg). This study used G*power (http://www.gpower.hhu.de/) to calculate the power. In this study, the effect size f = 0.25 was used because it was the large effect size based on the partial eta squared method (Cohen and Cohen, 1988). By this calculation, recruiting 30 healthy young adults could provide 90% power for using the repeated measure. These young adults were free from any neurological or musculoskeletal disorders. Also, they had no history of lower extremity injuries and no history of falls in the prior years. These young adults also needed to have a score of 52/56 or above on the Berg Balance Scale, indicating no balance impairment. An at-home visual test (visual chart, Ultrassist, Amazon, US) was also given to all participants. Participants needed to reach 20/20 vision level from 6 feet distance; otherwise, they were asked to wear their own glasses to reach 20/20 vision level. All abovementioned tests were performed by these participants 1 week before the day of data collection. Also, participants were excluded from this study if they had a score above zero on the dizziness handicap inventory, indicating potential vestibular deterioration. Before the data collection began, each participant signed an informed consent. This study followed the guidelines and regulations of the University of Nebraska Medical Center Institutional Review Board that approved this study (IRB# 340-10-FB).

### Experimental Protocol

**FIGURE 1:**
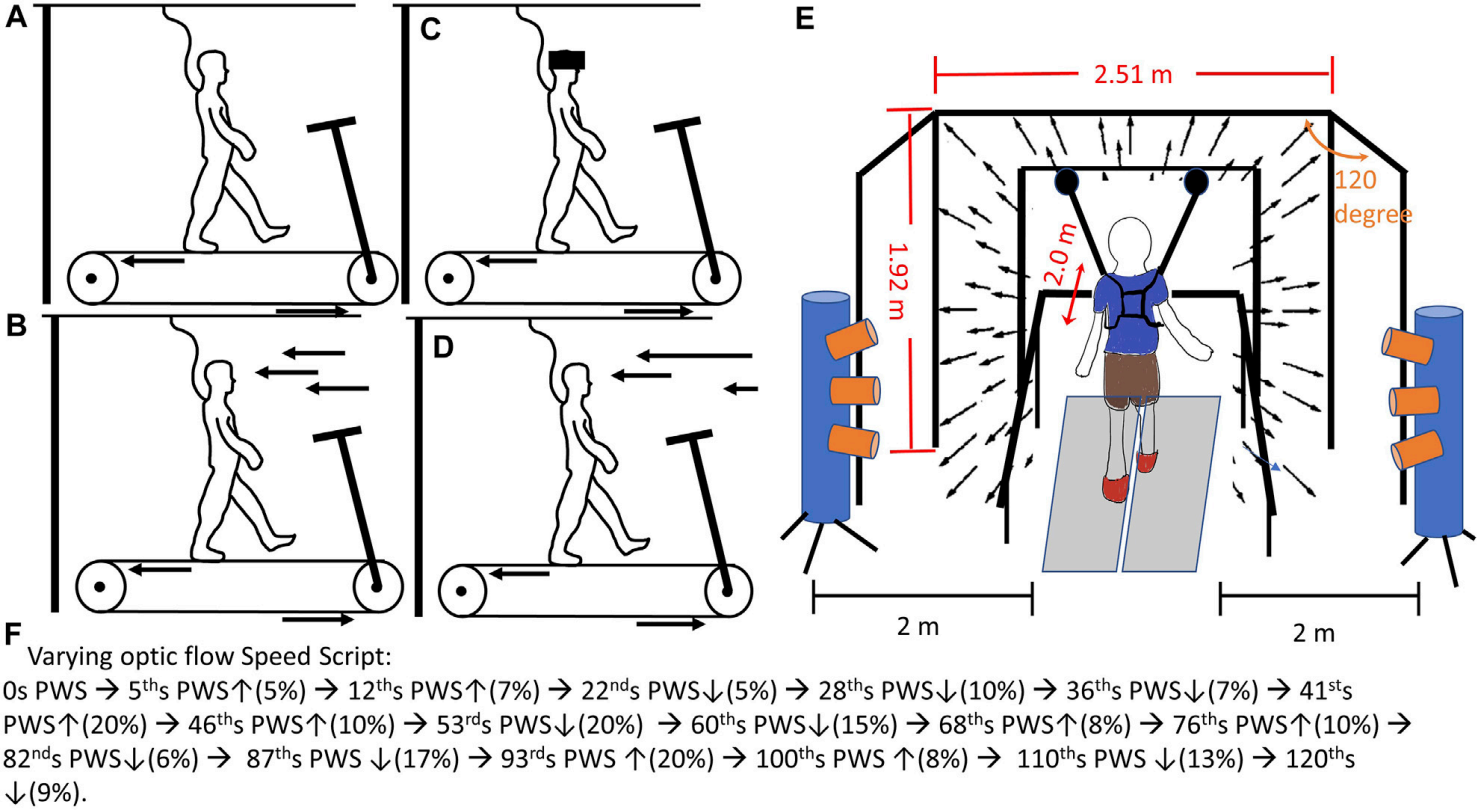
The experimental diagram: **(A)** walking on the treadmill without optic flow (noOptic), 35; **(B)** walking on the treadmill with the optic flow and the speed of optic flow was matched to treadmill speed (Optic); **(C)** walking on the treadmill with goggle, which reduced the visual capabilities for participants (Vre); **(D)** walking on the treadmill under pseudo-random visual perturbations (Vpe). This pseudo-random was made by the script: ↑ represented speed up % of preferred walking speed, and ↓ represented speed down % of preferred walking speed. **(E)** This virtual reality provided an optic flow speed matched/mismatched to participants’ preferred walking speed coding by Python using the WorldViz LLC graphics library (Santa Barbara, CA, United States). The optic flow was projected by three commercial projection systems (Optoma TX 774, Optoma Technology Inc., Milpitas, CA). Each screen was of 2.51 m width and 1.72 m height. The front screen was placed 2 m away from the treadmill. The side screen placed 120° against the front screen. The two motion capture cylinders with three lenses for each cylinder (Optotrak Certus, Northern Digital Inc., Waterloo, Canada) were placed 2 m away from the treadmill on the side. This optimal range was set based on the Optotrak Certus User Guide (https://tsgdoc.socsci.ru.nl/images/e/eb/Optotrak_Certus_User_Guide_rev_6%28IL-1070106%29.pdf) for accurately capturing the infrared light emitting diode markers placed on hip (greater trochanter), knee (lateral epicondyle), ankle (lateral malleolus), toe (2nd Metatarsal), and heel of each foot. The accuracy of this motion capture system was up to 0.1 mm and resolution 0.01 mm. This motion capture system was validated by Schmidt et al.’s study (Greenlee, 2017).

**FIGURE 2:**
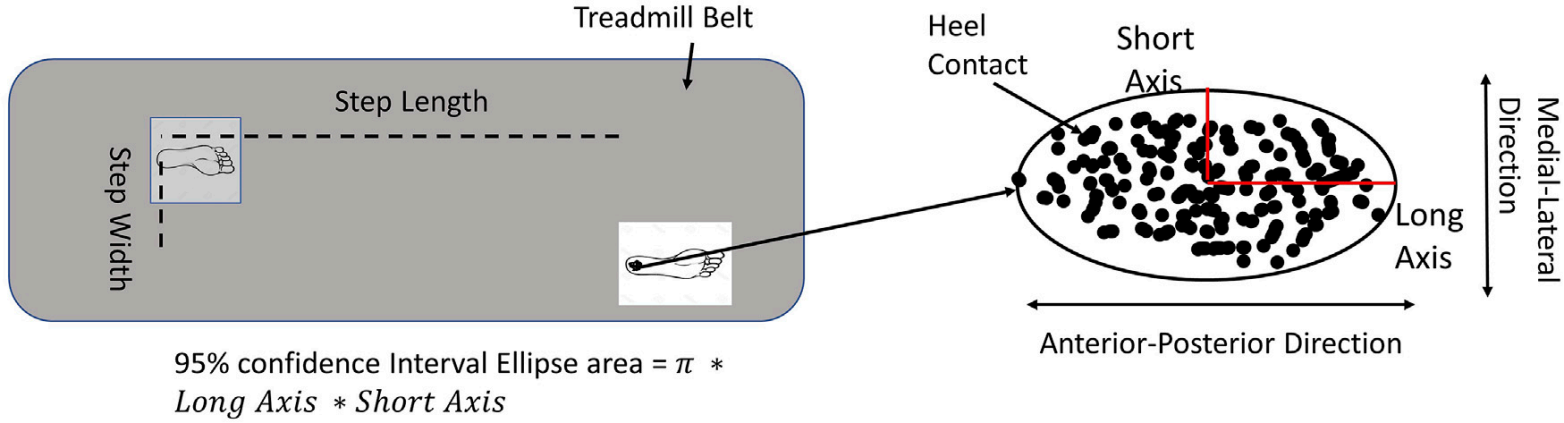
The data processing method. For the demands of active control, the step length variability and step width variability were used. For the types of active control, the 95% confidential interval ellipse area was calculated by each heel contact during initial 200 steps.

**FIGURE 3:**
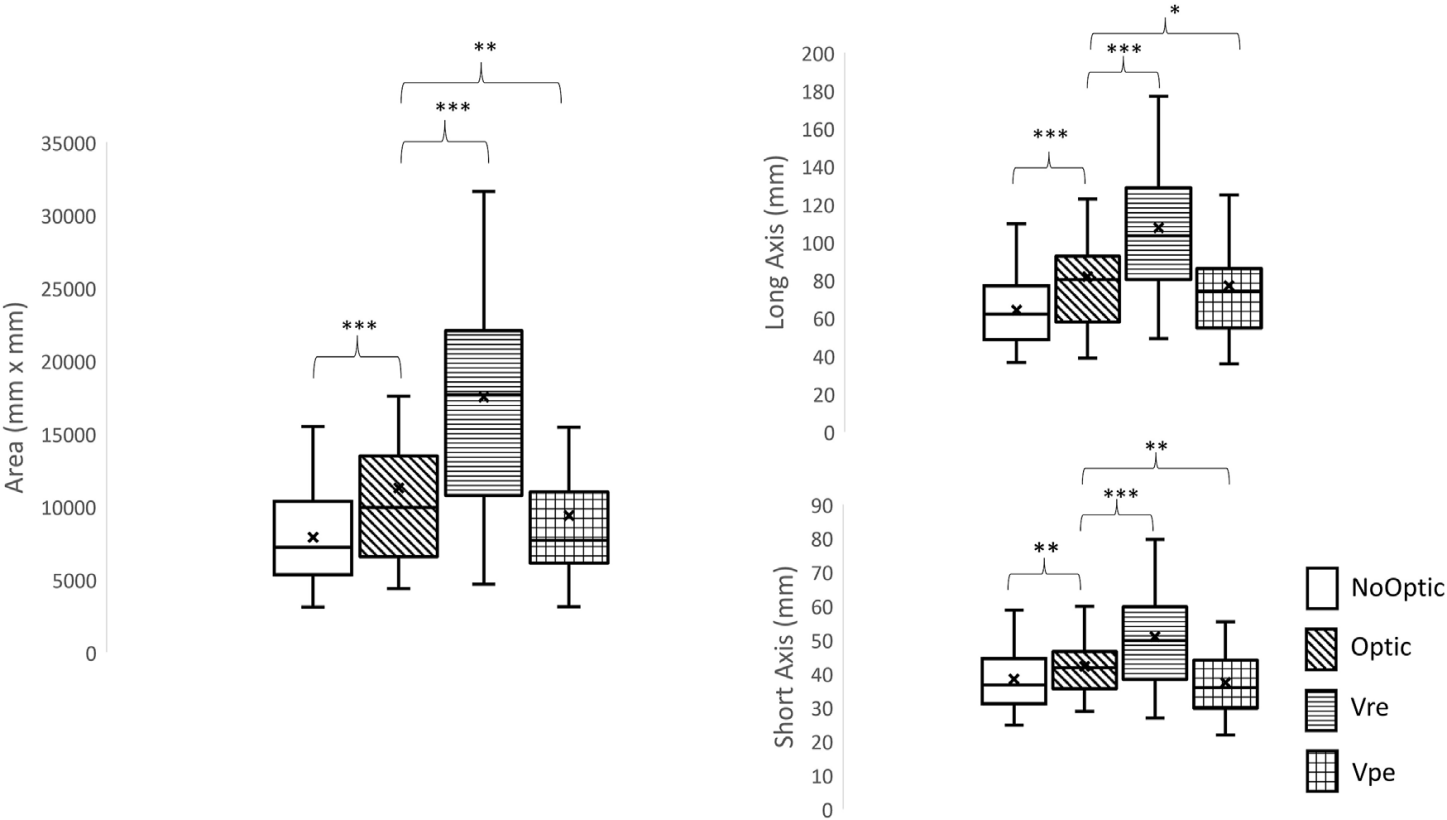
The ellipse area, the length of long axis and the length of short axis. *: significant difference compared to optic flow condition, *: *p* < 0.05, **: *p* < 0.01; ***: *p* < 0.001.

Prior to the data collection, the preferred walking speed (PWS) for each participant needed to be identified. This information can be found in Supplementary Material. The participant placed both feet on the side without touching the treadmill belt and held the handrail. An experimenter started the treadmill at 0.8 m/s. When the treadmill belt consistently ran at 0.8 m/s for 10 s, participants stepped on the treadmill belt while holding the handrail. When participants got used to walking on the treadmill, they were encouraged to release the handrail. An experimenter asked, “Is this walking speed comfortable for you, like walking around the grocery store?”. Based on the participant’s responses, the treadmill speed was adjusted by increasing or decreasing by 0.1 m/s, then participants continued walking for 20 s at the adjusted speed. The above procedure was repeated by an experimenter until the participant’s PWS was identified. Once the PWS was determined, the participants walked on the treadmill for 5 min to familiarize themselves with treadmill walking. For safety issues, participants could hold the handrail anytime if they felt unsteady. Also, participants had the right to terminate the data collection at any time if they felt uncomfortable. After the familiarization, a mandatory rest was given to participants for 2 min. Next, a total of four 2-min walking conditions were randomly assigned to participants (Figure 1): 1) walking without optic flow (NoOptic), participant walks with eye open, but optic flows were not projected to screens (Figure 1A), 2) walking with the optic flow (the treadmill speed was matched to optic flow speed, Optic) (Figure 1B), 3) walking under reduced visual capability (Vre) (Figure 1C), and 4) walking under perturbed optic flow (the treadmill speed was mismatched to optic flow speed, and the optic flow speed varied randomly between 80% and 120% of the treadmill speed, Vpe, Figure 1D). This 2-min interval was set to capture the step-to-step gait adjustments with/without optic flow assistance/perturbations (Hu and Chien, 2021). Also, it was worth mentioning that the Optic was defined as the control condition. Between each condition, a 2 min mandatory rest was given to participants to eliminate the learning effect from the prior condition (Chien et al., 2017). The average preferred walking speed was 1.48 ± 0.08 m/s.

### Experimental Setup

Participants walked within a virtual reality environment (Figure 1E). A motion capture (Optotak Certus, Northern Digital Inc., Waterloo, Canada) was used to track the trajectories of body landmarks as follows: hip (greater trochanter), knee (lateral epicondyle), ankle (lateral malleolus), toe (2nd metatarsal), and heel of each foot (Schmidt et al., 2009). An instrumented treadmill (Bertec Corp., Columbus, OH, United States) was used to record the ground reaction force at 100 Hz. Each walking condition lasted 2 minutes. For the condition of walking under perturbed optic flow, the pseudo-random optic flow speed sequence was set as following steps: 1) developing time intervals—randomly generating values between five and ten, repeatedly, until the sum of these values reached 120. In the current study, a total of seventeen time intervals were generated. 2) developing and assigning the values of speed into these seventeen time intervals—randomly generating a value between negative twenty and positive twenty and inserted this value into a time interval; however, if the sum of values from already generated and from the current generated exceeded negative twenty or positive twenty, the current value would be re-generated. These values of speed were the percentage of participants’ preferred walking speed. This set of speed range has been widely used to represent the comfortable walking speed from slow (80%) to fast (120%) pace (Jordan et al., 2007; Kang and Dingwell, 2008; Chien et al., 2015; Lee et al., 2021). The final sequence is shown in Figure 1F, and all the participants used this sequence. Additionally, none of the participants experienced walking within a virtual reality environment previously. For the condition of walking under low visual capacity, a reduced-light intensity goggle with car tinting vinyl was given to all participants. By wearing this reduced-light intensity goggle (MSA Safety Work, Pittsburgh, PA), the light intensity of 0.7 lx could be perceived for all participants (150 lx was the light intensity for a regular office). The light intensity was measured by the light meter once before the beginning of data collection when the probe of a light meter (Dr. Meter, support@drmeter.com) was placed between the lens of the goggle and the participants’ eyes. The ground reaction force was used to identify two critical gait events—heel contact and toe-off. The heel contact was defined by the frame in which the vertical component of ground reaction force reached 10 N and above and was sustained for 40 ms (Chien et al., 2014). On the other hand, the toe-off was defined by the frame in which the vertical component of ground reaction force dropped to 10 N and below and was sustained for 40 ms (Chien et al., 2014). The ground reaction force data and motion capture data were synchronized through the NDI First Principal software (Optotak Certus, Northern Digital Inc., Waterloo, Canada). The step length was defined as the length from the heel contact of one leg to the heel contact of the contralateral leg. Also, the step width was defined as the width from the heel contact of one leg to the heel contact of the contralateral leg. The gait variability was the coefficient of variation, which was suggested as a more appropriate measure of gait variability than using the standard deviation (Wurdeman and Stergiou, 2013).The spatial gait variabilities were suggested to identify the demands of active control (Bauby and Kuo, 2000; Hu and Chien, 2021). On the other hand, the 95% confidence interval of the ellipse was used to evaluate the pattern of the active control (Hu and Chien, 2021). To ensure safety, participants had to wear the vest, which was attached to a LiteGait harness system (Mobility Research, AZ, United States) when walking on the treadmill.

The 95% confidence ellipse area was calculated by the following formula (Prieto and Myklebust, 1993):

$$LongAxis={\left[F_{\alpha \left[2,n-2\right]}\left(S_{AP}^{2}+S_{ML}^{2}+D\right)\right]}^{1/2};ShortAxis={\left[F_{\alpha \left[2,n-2\right]}\left(S_{AP}^{2}+S_{ML}^{2}+D\right)\right]}^{1/2},$$

where $D={\left[{\left(S_{AP}^{2}+S_{ML}^{2}\right)}^{2}-4\left(S_{AP}^{2}S_{ML}^{2}-S_{AP,ML}^{2}\right)\right]}^{1/2}$ . $F_{\alpha \left[2,n-2\right]}$ is the F statistic at the confidence level of 1—a with n data points, S_AP_, and S_ML_ are the standard deviations of the anterior-posterior (AP, Long axis) and medial-lateral (ML, short axis) direction respectively, and S_AP_, _ML_ is the covariance.

Dependent variables were mean step length, mean step width, step length variability, step width variability, the 95% confidence interval of the ellipse area of heel contact locations, the long axis of the ellipse (heel contact locations in the anterior-posterior direction), and the short axis of the ellipse (heel contact locations in the medial-lateral direction, Figure 2) (Hu and Chien, 2021).

### Statistical Analysis

The Shapiro–Wilk normality test was used to test the normality of each dependent variable. If the alpha value of Shapiro–Wilk was larger than 0.05, a one-way repeated ANOVA measure was used to investigate the effect of different types of visual manipulations. Bonferroni corrections were used for post hoc pairwise comparisons for each dependent variable. If the alpha value of Shapiro–Wilk was smaller than 0.05, a Friedman test was used to investigate the effect of different types of visual manipulations. The Wilcoxon signed rank test was used for pairwise comparisons for each dependent value. The partial eta squared method was used to investigate the effect size (Cohen and Cohen, 1988).

## Results

### The Effect of Different Types of Visual Manipulations

The Friedman Tests demonstrated that significant effect of visual manipulations was found in the step length (χ^3^ = 47.68, *p* < 0.001), the step width (χ^3^ = 46.82, *p* < 0.001), the step length variability (χ^3^ = 64.36, *p* < 0.001), the step width variability (χ^3^ = 67.48, *p* < 0.001), the 95% confidence interval ellipse area (χ^3^ = 66.28, *p* < 0.001), the long axis of the ellipse (χ^3^ = 60.88, *p* < 0.001), and the short axis of the ellipse (χ^3^ = 43.72, *p* < 0.001).

1) Wilcoxon signed rank tests indicated that the significant larger step length variability (Z = −4.78, *p* < 0.001), larger step width variability (Z = −4.78, *p* < 0.001), larger ellipse area (Z = −4.53, *p* < 0.001), larger long axis of the ellipse (Z = −3.94, *p* < 0.001), and larger short axis of the ellipse (Z = −3.71, *p* < 0.001) were observed in the Vre condition than in the Optic condition.
2) Wilcoxon signed rank tests indicated that the significant larger step width variability (Z = −4.68, *p* < 0.001), smaller ellipse area (Z = −3.08, *p* = 0.002), shorter long axis of the ellipse (Z = −2.31, *p* = 0.021), and shorter short axis of the ellipse (Z = −3.36, *p* = 0.001) were observed in the Vpe condition than in the Optic condition.
3) Wilcoxon signed rank tests indicated that the significant larger step length variability (Z = −4.70, *p* < 0.001), larger step width variability (Z = −4.47, *p* < 0.001), smaller ellipse area (Z = -3.08, *p* = 0.002), shorter long axis of the ellipse (Z = −3,73, *p* < 0.001), and shorter short axis of the ellipse (Z = −2.97, *p* = 0.003) were observed in the noOptic condition than in the Optic condition.
4) Wilcoxon signed rank tests indicated that Optic-NoOptic: *p* < 0.001, Vre-NoOptic: *p* < 0.001, Vpe-NoOptic: *p* = 0.019, Vre-Optic: *p* < 0.001, Vpe-Optic: *p* = 0.002, Vpe-Vre: *p* < 0.001.

More details are shown in Table 1 and Figure 3.

**TABLE 1:** Mean and standard deviation of step length, step length variability, step width, step width variability, ellipse area, long axis of ellipse area, and short axis of ellipse area with comparisons.

	NoOptic	Optic	Vre	Vpe		
Step length (mm)	566.3 (64.4)	580.3 (66.4)	544.7 (83.2)	566.9 (66.4)		
Comparisons	Optic vs. NoOptic	Vre vs. NoOptic	Vpe vs. NoOptic	Vre vs. Optic	Vpe vs. Optic	Vpe vs. Vre
	Z = −4.25, *p* < 0.001	Z = −4.06, *p* < 0.001	Z = −0.28, *p* = 0.781	Z = −4.70, *p* < 0.001	Z = −4.17, *p* < 0.001	Z = −3.53, *p* < 0.001
	NoOpitc	Optic	Vre	Vpe		
Step length variability	4.47 (1.36)	3.67 (0.82)	7.33 (2.09)	3.92 (1.21)		
Comparisons	Optic vs. NoOptic	Vre vs. NoOptic	Vpe vs. NoOptic	Vre vs. Optic	Vpe vs. Optic	Vpe vs. Vre
	Z = −4.70, *p* < 0.001	Z = −4.56, *p* < 0.001	Z = −2.73, *p* = 0.006	Z = −4.78, *p* < 0.001	Z = −0.422, *p* = 0.67	Z = −4.78, *p* < 0.001
	NoOpitc	Optic	Vre	Vpe		
Step width (mm)	118.8 (33.6)	113.4 (35.6)	127.5 (32.8)	121.3 (34.5)		
Comparisons	Optic vs. NoOptic	Vre vs. NoOptic	Vpe vs. NoOptic	Vre vs. Optic	Vpe vs. Optic	Vpe vs. Vre
	Z = −3.06, *p* = 0.002	Z = −4.06, *p* < 0.001	Z = −1.20, *p* = 0.229	Z = −4.78, *p* < 0.001	Z = −3.45, *p* = 0.001	Z = −3.56, *p* < 0.001
	NoOptic	Optic	Vre	Vpe		
Step width variability	15.68 (3.23)	13.21 (3.28)	20.09 (4.42)	15.33 (3.72)		
Comparisons	Optic vs. NoOptic	Vre vs. NoOptic	Vpe vs. NoOptic	Vre vs. Optic	Vpe vs. Optic	Vpe vs. Vre
	Z = −4.47, *p* < 0.001	Z = −4.62, *p* < 0.001	Z = −0.89, *p* = 0.37	Z = −4.78, *p* < 0.001	Z = −4.68, *p* < 0.001	Z = −4.72, *p* < 0.001
	NoOptic	Optic	Vre	Vpe		
Ellipse area (mm x mm)	7886.22 (3379.22)	11260.67 (6162.81)	17504.64 (8521.84)	9378.68 (5053.58)		
Comparisons	Optic vs. NoOptic	Vre vs. NoOptic	Vpe vs. NoOptic	Vre vs. Optic	Vpe vs. Optic	Vpe vs. Vre
	Z = −4.29, *p* < 0.001	Z = −4.78, *p* < 0.001	Z = −2.355, *p* = 0.019	Z = −4.54, *p* < 0.001	Z = −3.08, *p* = 0.002	Z = −4.78, *p* < 0.001
	NoOptic	Optic	Vre	Vpe		
Long axis of area (mm)	64.16 (18.96)	81.92 (31.52)	107.71 (42.92)	77.11 (29.78)		
Comparisons	Optic vs. NoOptic	Vre vs. NoOptic	Vpe vs. NoOptic	Vre vs. Optic	Vpe vs. Optic	Vpe vs. Vre
	Z = −3.73, *p* < 0.001	Z = −4.76, *p* < 0.001	Z = −3.01, *p* = 0.003	Z = −3.94, *p* < 0.001	Z = −2.31, *p* = 0.002	Z = −4.78, *p* < 0.001
	NoOptic	Optic	Vre	Vpe		
Short axis of area (mm)	38.33 (9.226)	42.16 (8.83)	50.91 (15.01)	37.42 (9.02)		
Comparisons	Optic vs. NoOptic	Vre vs. NoOptic	Vpe vs. NoOptic	Vre vs. Optic	Vpe vs. Optic	Vpe vs. Vre
	Z = −2,97, *p* = 0.003	Z = −4.04, *p* < 0.001	Z = −0.89, *p* = 0.371	Z = −3.71, *p* < 0.001	Z = −3.363, *p* = 0.001	Z = −4.78, *p* < 0.001

## Discussion

This study aimed to 1) build an “unexpected-enough” visual-perturbed paradigm for future sensorimotor training and 2) identify the demands and patterns in the active control under different types of visual perturbations. The results confirmed our hypotheses that 1) applying the Vre or the Vpe in the AP direction affected the gait in both AP and ML directions, indicating that the impact of visual perturbations were stronger in the current study than in previous studies, and 2) using the extended active control hypothesis indeed differentiated the patterns of active control among four visual-perturbed conditions.

### Reduced Visual Capacity Perturbation Increased the Demand in Active Control Most

Unsurprisingly, the results revealed a significant increase in the demand of active control in the Optic condition compared to the Vre condition in this current study. A study showed that healthy young adults reduced 34% of their walking speeds during overground walking when the visual system was deprived (Hallemans et al., 2009). However, it was not possible to slow down the walking speed while walking on the treadmill with reduced visual capacity. Therefore, this reduced visual capability condition might be compensated for by the vestibular system and somatosensory systems. For the vestibular system, the vestibulospinal tract has been thought as a crucial role in executing the voluntary forward steps and mediating postural adjustments to maintain balance (Bent et al., 2002). Subtle movements of the body are detected by the vestibular sensory neurons, and motor commands to counteract these movements are sent through the vestibulospinal tracts to appropriate muscle groups. Specifically, the lateral vestibulospinal tract activated the antigravity muscles to compensate for the postures from the tilts of the body. A study even suggests that repeating the progress of sensory reweighting from visual to the vestibular system would contribute to the correct facilitation of anticipatory postural adjustment (Nallegowda et al., 2004), further enhancing the feed-forward mechanisms prior to voluntary movements (Tramontano et al., 2016). Secondly, the somatosensory system plays a critical role in providing the feedback information because it is the only part of the body in contact with the ground. In the current study, the level of active control was the highest in the Vre than in other conditions, indicating that healthy young people actively used exploratory strategies (larger area) to interact with the treadmill to maintain the walking stability in this Vre condition. Therefore, the purpose of this visual-reduced training could train both feedback and feed-forward capabilities simultaneously to handle an unknown environment, such as walking with malfunctioned otoliths under low-gravity. This blindfolded sensorimotor training has been approved to enhance the balance and locomotor control in healthy young adults (Cha et al., 2016; Lin et al., 2021), in patients with Parkinson’s Disease (Tramontano et al., 2016; Bonnì et al., 2019), and in patients with unilateral lower-limb amputees (Vrieling et al., 2008). In the current study, we suggest this reduced visual capability sensorimotor training most to future astronauts; it costs less (only required a treadmill and a goggle with car tinting vinyl) and had the strongest impact among other visual manipulations in the current study. However, we do not suggest this training to patients with neurological or musculoskeletal disorders due to the safety issues because, in our other not-yet-published studies, we indeed observed some trips on the treadmill with reduced visual capability in healthy older adults while wearing a harness. The consequence was that these healthy older adults either did not want to continue walking on the treadmill or changed their gait pattern dramatically (asked for slower belt speed and walked very carefully within each step).

### Pseudo-Random Visual Perturbation Also Increased Demands of Active Control in the Medial-Lateral Direction

Surprisingly, an increase in the step width variability but a decrease in the ellipse area was observed in Vpe compared to the Optic condition in this current study, indicating 1) the significant increase in demand of active control, particularly in the medial-lateral direction, and 2) a decrease in the ellipse area was also a sign of increase in active control, inferring the different patterns of active control. This result was different than previous studies (O’Connor and Kuo, 2009), that manipulates the timing and the speed of optic flow in the anterior-posterior direction did not increase the demands in active control in the medial-lateral direction. We speculated that the pseudo-random visual perturbation in the current study might disrupt the depth information during walking. The visual process depended on two pathways: the ventral pathway was for perceiving and identifying the visual input (from the primary visual cortex, V1, to the inferior temporal lobe), and the dorsal pathway was to use visual information for guiding real-time action (projecting from V1 to the posterior parietal lobe) (Goodale, 2017). Additionally, the dorsal pathway primarily depends on binocular information to identify the cues to depth (Mon-Williams and Tresilian, 1999). If the binocular information was perturbed/impaired, actions in the virtual environment might rely greatly on ventral input, resulting in movement inefficiency—unable to manipulate body position, maintain balance, and execute movement (Loftus et al., 2004). In the current study, the pseudo-random visual perturbations may generate visual conflicts in the perception of depth due to the random alternations in the rhythm and the speed of optic flow (Tresilian et al., 1999), leading to unreliable binocular information and causing a great reliance on ventral processing (Marotta et al., 1998). This result might cause movement inefficiency eventually, and this was why we observed the abovementioned alternations, indicating greater active control in the medial-lateral direction in the Vpe than in the Optic condition. Based on the sensorimotor training point of view, this Vpe was relatively safe in comparison with the Vre. This study tends to suggest this Vpe training for patients with neurological or musculoskeletal disorders.

### Providing the Optic Flow During Treadmill Walking Increased the Active Control Than Walking Without Optic Flow

Treadmill gait training has numerous advantages compared with overground gait training, as follows: 1) treadmill training can be completed in a small area, 2) larger volume of steps can be achieved, 3) walking speed can be well-controlled and assisted by physical therapists, and 4) the body weight can be supported by a harness. For instance, for patients with spinal cord injury (SCI), a study showed that treadmill gait training is not better than overground gait training in patients with SCI (Wolpaw, 2006). One possible limitation of treadmill gait training compared to overground gait training was the lack of visual perception during walking. Also, it has been reported that participants walk approximately 17% slower on the treadmill than their corresponding overground preferred speed when the digital speed display of the treadmill is blinded in both young and older adults (Marsh et al., 2006; Dal et al., 2010). Moreover, many previous studies have found differences in the spatial-temporal gait parameters, such as wider step width, shorter step length, and greater gait variability (Murray et al., 1985; Alton et al., 1998; Riley et al., 2007; Watt et al., 2010) during treadmill walking than during overground walking. It may be that when walking on the treadmill, participants almost keep stationary in a limited space. Therefore, the self-reference (the self-position related to the environment) is barely changed, and the level of the vision system may not be highly used in real-time compared to overground walking. Instead, the sensation from plantar somatosensory may be heavily used to adjust self-speed to match the treadmill and to cause visual-somatosensory conflicts (Greenlee, 2017).On the other hand, when walking overground, both vision and somatosensory systems are heavily integrated to identify the self-reference for path identification. Thus, to enhance the treadmill gait training, synchronizing the visual and somatosensory systems for controlling the self-reference might open an alternative solution by using optic flow matched to participants’ walking speed. In the current study, we observed decrement in the gait variability and step width but increase in step length and ellipse area, while the optic flow was provided, which supported that implementing optic flow enhanced the control of self-conference. Additionally, this enhancement of control of self-conference demanded active control. Therefore, increasing the demand for active control might be the key to future training.

### Using 95% Confidence Interval Ellipse Can Identify the Patterns of Different Types of Visual Perturbations

Step length variability and step width variability can only identify the levels of demand in active control; however, these two measures cannot assess the pattern in active control. Hu and Chien (Hu and Chien, 2021) proposed a novel measure to categorize the patterns in active control by using the 95% confidence interval ellipse area. This study attempted to extend this measure to identify the patterns from four different types of visual-perturbed environments. From our observations (Supplementary Material S2), four different ellipse areas were found and it clearly demonstrated that four different strategies were used under four different types of visual perturbations because the pairwise comparisons showed that all conditions were significantly different than each other. Specifically, when Vre condition was given to participants, the ellipse area was the largest, indicating that the young adults were to explore the environment and find optimal foot placements to maintain balance in comparison with the Optic condition. On the other hand, when the Vpe condition was given to participants, the ellipse area became smaller compared to the Optic condition, referring to those young adults who used a conservative strategy to limit their foot placements to prevent trips. To our best knowledge, this was the first study to use this measure to identify the patterns of active control from different types of visual perturbations. This measure could be used to determine the patterns of locomotor behaviors under sensorimotor training.

### Limitation

The limitation of this study was that the visual perturbations in the medial-lateral direction were not provided; therefore, whether pseudo-random visual perturbations in the medial-lateral direction would affect the demands and patterns in active control in both anterior-posterior and medial-lateral directions remains unknown. The second limitation was that motor learning was not studied in this current study. Future studies need to focus on these two fields.

## Conclusion

This was the first study using the extended active control hypothesis to identify the demands and patterns under different types of visual perturbations. Understanding the abovementioned demands and patterns of active control would assist patients/astronauts to build a sensorimotor training protocol. This study tended to suggest the Vre sensorimotor training for future astronauts and Vpe for patients with neuromuscular diseases (Schmidt et al., 2009; Carriot et al., 2021).

## Data Availability

The original contributions presented in the study are included in the article/Supplementary Material; further inquiries can be directed to the corresponding author.

## References

[B1] Alton F. Baldey L. Caplan S. Morrissey M. C. (1998). A Kinematic Comparison of Overground and Treadmill Walking. Clin. Biomech. 13 (6), 434–440. 10.1016/s0268-0033(98)00012-6 11415818

[B2] Bauby C. E. Kuo A. D. (2000). Active Control of Lateral Balance in Human Walking. J. biomechanics 33 (11), 1433–1440. 10.1016/s0021-9290(00)00101-9 10940402

[B3] Bent L. R. Inglis J. T. McFadyen B. J. (2002). Vestibular Contributions across the Execution of a Voluntary Forward Step. Exp. Brain Res. 143 (1), 100–105. 10.1007/s00221-001-0967-7 11907695

[B4] Berard J. R. Fung J. Lamontagne A. (2011). Evidence for the Use of Rotational Optic Flow Cues for Locomotor Steering in Healthy Older Adults. J. Neurophysiology 106 (3), 1089–1096. 10.1152/jn.00277.2011 21653718

[B5] Bloomberg J. J. Mulavara A. P. (2003). Changes in Walking Strategies after Spaceflight. IEEE Eng. Med. Biol. Mag. 22 (2), 58–62. 10.1109/memb.2003.1195697 12733460

[B6] Bonnì S. Ponzo V. Tramontano M. Martino Cinnera A. Caltagirone C. Koch G. (2019). Neurophysiological and Clinical Effects of Blindfolded Balance Training (BBT) in Parkinson's Disease Patients: a Preliminary Study. Eur. J. Phys. Rehabil. Med. 55 (2), 176–182. 10.23736/S1973-9087.18.05126-2 29745627

[B7] Caballero C. Davids K. Heller B. Wheat J. Moreno F. J. (2019). Movement Variability Emerges in Gait as Adaptation to Task Constraints in Dynamic Environments. Gait posture 70, 1–5. 10.1016/j.gaitpost.2019.02.002 30771594

[B8] Carriot J. Mackrous I. Cullen K. E. (2021). Challenges to the Vestibular System in Space: How the Brain Responds and Adapts to Microgravity. Front. Neural Circuits 15, 760313. 10.3389/fncir.2021.760313 34803615 PMC8595211

[B9] Cha H. G. Lee B. J. Lee W. H. (2016). The Effects of Horse Riding Simulation Exercise with Blindfolding on Healthy Subjects' Balance and Gait. J. Phys. Ther. Sci. 28 (11), 3165–3167. 10.1589/jpts.28.3165 27942142 PMC5140822

[B10] Chien J. H. Eikema D.-J. A. Mukherjee M. Stergiou N. (2014). Locomotor Sensory Organization Test: a Novel Paradigm for the Assessment of Sensory Contributions in Gait. Ann. Biomed. Eng. 42 (12), 2512–2523. 10.1007/s10439-014-1112-7 25224076 PMC4241158

[B11] Chien J. H. Yentes J. Stergiou N. Siu K. C. (2015). The Effect of Walking Speed on Gait Variability in Healthy Young, Middle-Aged and Elderly Individuals. J. Phys. Act. Nutr. Rehabil. Available at: http://www.panr.com.cy/index.php/article/the-effect-of-walking-speed-on-gait-variability-in-healthy-young-middle-aged-and-elderly-individuals/ . PMC4768759 26929929

[B12] Chien J. H. Mukherjee M. Kent J. Stergiou N. (2017). Mastoid Vibration Affects Dynamic Postural Control during Gait in Healthy Older Adults. Sci. Rep. 7, 41547. 10.1038/srep41547 28128341 PMC5269701

[B13] Cohen J. (1988). “Some Issues in Power Analysis,” in Statistical Power Analysis for the Behavior Sciences. Editor Cohen J.. 2nd ed. (Lawrence Erlbaum Associate), 531–537.

[B14] Courtine G. Papaxanthis C. Pozzo T. (2002). Prolonged Exposure to Microgravity Modifies Limb Endpoint Kinematics during the Swing Phase of Human Walking. Neurosci. Lett. 332 (1), 70–74. 10.1016/s0304-3940(02)00909-6 12377387

[B15] Dal U. Erdogan T. Resitoglu B. Beydagi H. (2010). Determination of Preferred Walking Speed on Treadmill May Lead to High Oxygen Cost on Treadmill Walking. Gait posture 31 (3), 366–369. 10.1016/j.gaitpost.2010.01.006 20129785

[B16] Davids K. Glazier P. Ara??jo D. Bartlett R. (2003). Movement Systems as Dynamical Systems. Sports Med. 33 (4), 245–260. 10.2165/00007256-200333040-00001 12688825

[B17] Eikema D. J. A. Chien J. H. Stergiou N. Myers S. A. Scott-Pandorf M. M. Bloomberg J. J. (2016). Optic Flow Improves Adaptability of Spatiotemporal Characteristics during Split-Belt Locomotor Adaptation with Tactile Stimulation. Exp. Brain Res. 234 (2), 511–522. 10.1007/s00221-015-4484-5 26525712 PMC4732903

[B18] Glasauer S. Amorim M. A. Bloomberg J. J. Reschke M. F. Peters B. T. Smith S. L. (1995). Spatial Orientation during Locomotion [correction of Locomation] Following Space Flight. Acta Astronaut. 36 (8-12), 423–431. 10.1016/0094-5765(95)00127-1 11540973

[B19] Goodale M. A. (2017). “Duplex Vision,” in The Blackwell Companion to Consciousness (New York: Wiley), 648–661. 10.1002/9781119132363.ch46

[B20] Greenlee M. W. (2017). Self-Motion Perception: Ups and Downs of Multisensory Integration and Conflict Detection. Curr. Biol. 27 (18), R1006–R1007. 10.1016/j.cub.2017.07.050 28950080

[B21] Hallemans A. Beccu S. Van Loock K. Ortibus E. Truijen S. Aerts P. (2009). Visual Deprivation Leads to Gait Adaptations that Are Age- and Context-specific: I. Step-Time Parameters. Gait posture 30 (1), 55–59. 10.1016/j.gaitpost.2009.02.018 19342241

[B22] Hu J. Chien J. H. (2021). Aging Affects the Demands and Patterns in Active Control under Different Sensory-Conflicted Conditions. Front. Aging Neurosci. 13, 742035. 10.3389/fnagi.2021.742035 34803656 PMC8602863

[B23] Jordan K. Challis J. H. Newell K. M. (2007). Walking Speed Influences on Gait Cycle Variability. Gait Posture 26 (1), 128–134. 10.1016/j.gaitpost.2006.08.010 16982195

[B24] Kang H. G. Dingwell J. B. (2008). Effects of Walking Speed, Strength and Range of Motion on Gait Stability in Healthy Older Adults. J. Biomechanics 41 (14), 2899–2905. 10.1016/j.jbiomech.2008.08.002 PMC9135052 18790480

[B25] Lee M. Youm C. Noh B. Park H. (2021). Low Composite Functional Movement Screen Score Associated with Decline of Gait Stability in Young Adults. PeerJ 9, e11356. 10.7717/peerj.11356 33987024 PMC8092110

[B26] Lin Y. Mukherjee M. Stergiou N. Chien J. H. (2021). Using Mastoid Vibration Can Detect the Uni/bilateral Vestibular Deterioration by Aging during Standing. J. Vestib. Res. Equilib. Orientat. 32, 145–154. Advance online publication. 10.3233/VES-210042 34180442

[B27] Loftus A. Servos P. Goodale M. A. Mendarozqueta N. Mon-Williams M. (2004). When Two Eyes Are Better Than One in Prehension: Monocular Viewing and End-point Variance. Exp. Brain Res. 158 (3), 317–327. 10.1007/s00221-004-1905-2 15164152

[B28] Marotta J. J. DeSouza J. F. X. Haffenden A. M. Goodale M. A. (1998). Does a Monocularly Presented Size-Contrast Illusion Influence Grip Aperture? Neuropsychologia 36 (6), 491–497. 10.1016/s0028-3932(97)00154-1 9705058

[B29] Marsh A. P. Katula J. A. Pacchia C. F. Johnson L. C. Koury K. L. Rejeski W. J. (2006). Effect of Treadmill and Overground Walking on Function and Attitudes in Older Adults. Med. Sci. sports Exerc. 38 (6), 1157–1164. 10.1249/01.mss.0000222844.81638.35 16775558

[B30] McAndrew Young P. M. Wilken J. M. Dingwell J. B. (2012). Dynamic Margins of Stability during Human Walking in Destabilizing Environments. J. biomechanics 45 (6), 1053–1059. 10.1016/j.jbiomech.2011.12.027 PMC3321251 22326059

[B31] Miller C. A. Peters B. T. Brady R. R. Richards J. R. Ploutz-Snyder R. J. Mulavara A. P. (2010). Changes in Toe Clearance during Treadmill Walking after Long-Duration Spaceflight. Aviat. space Environ. Med. 81 (10), 919–928. 10.3357/asem.2680.2010 20922883

[B32] Mon-Williams M. Tresilian J. R. (1999). Some Recent Studies on the Extraretinal Contribution to Distance Perception. Perception 28 (2), 167–181. 10.1068/p2737 10615458

[B33] Murray M. P. Spurr G. B. Sepic S. B. Gardner G. M. Mollinger L. A. (1985). Treadmill vs. Floor Walking: Kinematics, Electromyogram, and Heart Rate. J. Appl. physiology 59 (1), 87–91. 10.1152/jappl.1985.59.1.87 4030579

[B34] Nallegowda M. Singh U. Handa G. Khanna M. Wadhwa S. Yadav S. L. (2004). Role of Sensory Input and Muscle Strength in Maintenance of Balance, Gait, and Posture in Parkinson???s Disease. Am. J. Phys. Med. rehabilitation 83 (12), 898–908. 10.1097/01.phm.0000146505.18244.43 15624568

[B35] O'Connor S. M. Kuo A. D. (2009). Direction-dependent Control of Balance during Walking and Standing. J. neurophysiology 102 (3), 1411–1419. 10.1152/jn.00131.2009 19553493 PMC2746770

[B36] Prieto T. E. Myklebust J. B. (1993). Measures of Postural Sway. Clin. Pharmacol. Ther. 54 (2), 228. 10.1038/clpt.1993.134 8257503

[B37] Prokop T. Schubert M. Berger W. (1997). Visual Influence on Human Locomotion Modulation to Changes in Optic Flow. Exp. Brain Res. 114 (1), 63–70. 10.1007/pl00005624 9125452

[B38] Riley P. O. Paolini G. Della Croce U. Paylo K. W. Kerrigan D. C. (2007). A Kinematic and Kinetic Comparison of Overground and Treadmill Walking in Healthy Subjects. Gait posture 26 (1), 17–24. 10.1016/j.gaitpost.2006.07.003 16905322

[B39] Roller C. A. Cohen H. S. Kimball K. T. Bloomberg J. J. (2001). Variable Practice with Lenses Improves Visuo-Motor Plasticity. Cognitive Brain Res. 12 (2), 341–352. 10.1016/s0926-6410(01)00077-5 11587905

[B40] Schmidt J. Berg D. R. Ploeg H. L. Ploeg L. (2009). Precision, Repeatability and Accuracy of Optotrak Optical Motion Tracking Systems. Int. J. Exp. Comput. Biomechanics 1 (1), 114–127. 10.1504/ijecb.2009.022862

[B41] Tramontano M. Bonnì S. Martino Cinnera A. Marchetti F. Caltagirone C. Koch G. (2016). Blindfolded Balance Training in Patients with Parkinson's Disease: A Sensory-Motor Strategy to Improve the Gait. Parkinson's Dis. 2016, 7536862. 10.1155/2016/7536862 26977334 PMC4763005

[B42] Tresilian J. R. Mon-Williams M. Kelly B. M. (1999). Increasing Confidence in Vergence as a Cue to Distance. Proc. R. Soc. Lond. B 266 (1414), 39–44. 10.1098/rspb.1999.0601 PMC1689642 10081157

[B43] Vrieling A. H. van Keeken H. G. Schoppen T. Otten E. Hof A. L. Halbertsma J. P. K. (2008). Balance Control on a Moving Platform in Unilateral Lower Limb Amputees. Gait posture 28 (2), 222–228. 10.1016/j.gaitpost.2007.12.002 18207407

[B44] Watt J. R. Franz J. R. Jackson K. Dicharry J. Riley P. O. Kerrigan D. C. (2010). A Three-Dimensional Kinematic and Kinetic Comparison of Overground and Treadmill Walking in Healthy Elderly Subjects. Clin. Biomech. 25 (5), 444–449. 10.1016/j.clinbiomech.2009.09.002 20347194

[B45] Wolpaw J. R. (2006). Treadmill Training after Spinal Cord Injury: Good but Not Better. Neurology 66 (4), 466–467. 10.1212/01.wnl.0000203915.14930.b4 16505294

[B46] Wood S. J. Loehr J. A. Guilliams M. E. (2011). Sensorimotor Reconditioning during and after Spaceflight. NeuroRehabilitation 29 (2), 185–195. 10.3233/nre-2011-0694 22027081

[B47] Wurdeman S. R. Stergiou N. (2013). Temporal Structure of Variability Reveals Similar Control Mechanisms during Lateral Stepping and Forward Walking. Gait posture 38 (1), 73–78. 10.1016/j.gaitpost.2012.10.017 23245640

[B48] Young L. R. Shelhamer M. (1990). Microgravity Enhances the Relative Contribution of Visually-Induced Motion Sensation. Aviat. Space Environ. Med. 61 (6), 525–530. 2369392

