# Supplementary figures and images for: Different Types of Visual Perturbation Induced Different Demands and Patterns in Active Control: Implication for Future Sensorimotor Training
Source: Front Physiol. 2022 Jul 13;13:919816. doi: 10.3389/fphys.2022.919816 (PMC9325964; doi:10.3389/fphys.2022.919816)

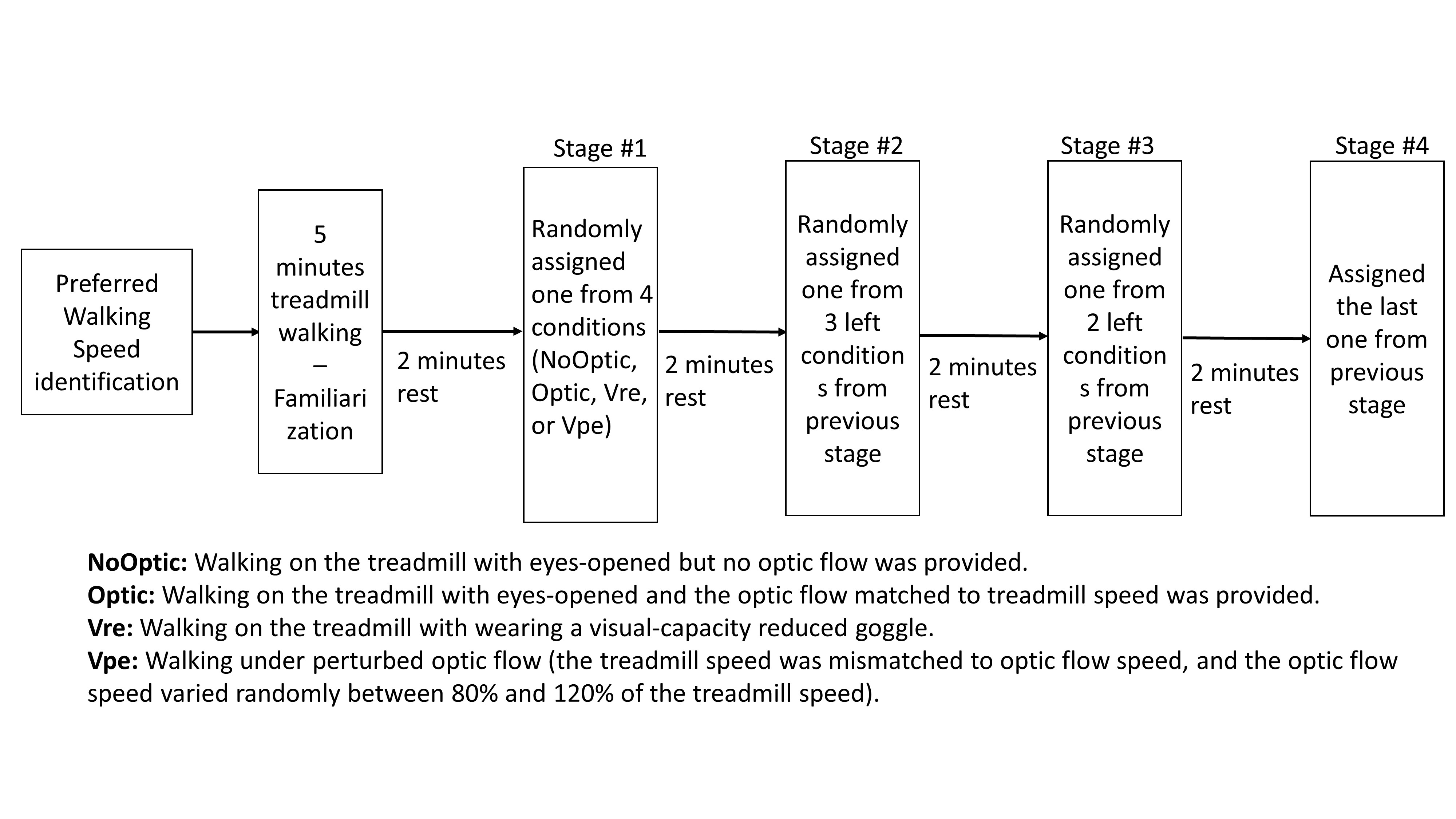

Supplement: Supplementary file 1 [file Image1.JPEG]

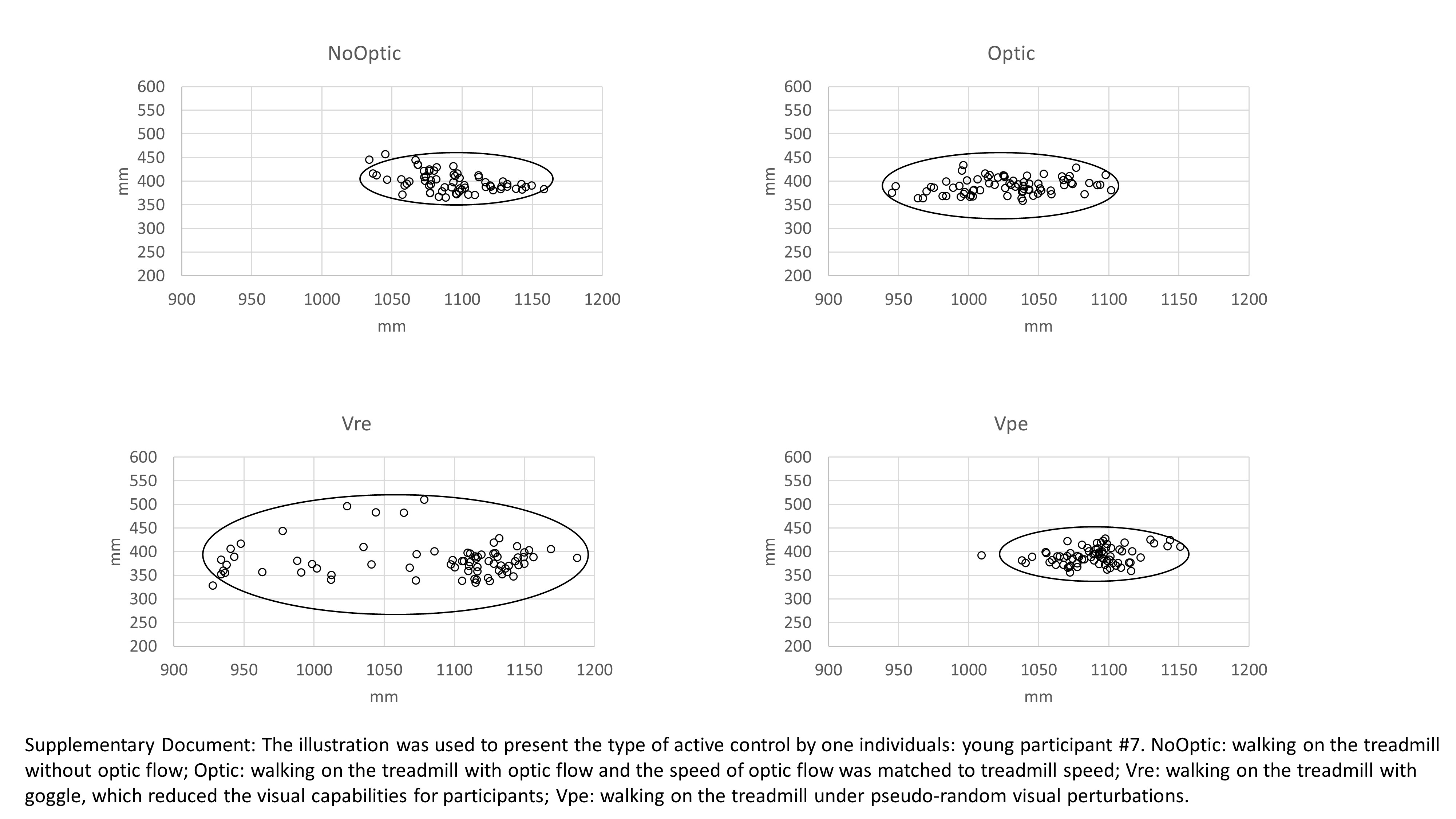

Supplement: Supplementary file 2 [file Image2.JPEG]
